# Trauma and chorioretinal shockwave injury from intra‐orbital foreign body

**DOI:** 10.1002/ccr3.8360

**Published:** 2023-12-27

**Authors:** Mehrdad Motamed Shariati, Naser Sahraei, Mahshid Sadeghi Kakhki

**Affiliations:** ^1^ Eye Research Center Mashhad University of Medical Sciences Mashhad Iran

**Keywords:** chorioretinal shockwave injury, chorioretinitis sclopetaria, intra‐orbital foreign body, ocular trauma

## Abstract

High‐velocity projectile trauma could lead to intra‐orbital foreign body and concomitant chorioretinal shockwave injury in the absence of open‐globe injury. Management depends on the types, size, and location of foreign bodies.

## Graphical abstract

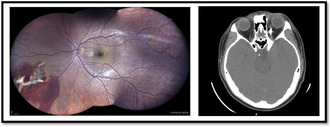

## 1 CASE PRESENTATION

**FIGURE 1:**
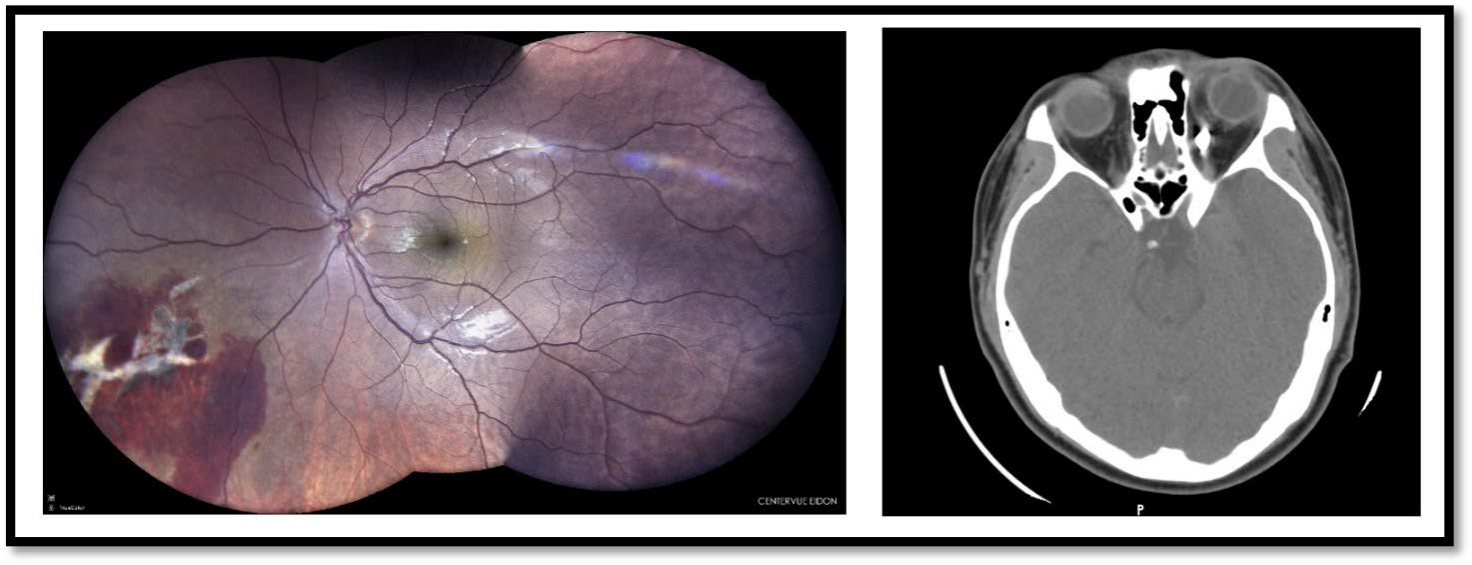
Traumatic chorioretinal shockwave injury and intra‐orbital foreign body.

A 15‐year‐old man was referred to our clinic with ocular projectile trauma of his left eye (LE) caused by hammering. Ophthalmic examination revealed lower and upper lid swelling, severe chemosis, and subconjunctival hemorrhage. The patient's uncorrected visual acuity (UCVA) was 10/10, and the relative afferent pupillary defect (RAPD) was negative. Ocular motility was limited in adduction in the LE. Inferior vitreous hemorrhage, intraretinal hemorrhage, and inferior‐nasal chorioretinal shockwave injury with choroidal rupture (chorioretinitis sclopetaria) were apparent in the fundus examination (Figure 1, left image). The orbital CT scan of the patient revealed a metallic foreign body in the medial rectus muscle near the optic nerve (Figure 1, right image). We found no sign of open globe injury in diagnostic peritomy. The patient is under periodic ophthalmic examinations.

## 2 DISCUSSION

Chorioretinitis sclopetaria is a rare trauma sequel that defines the choroid and retina rupture due to a blunt or a high‐velocity object. The projectile trauma can cause a shockwave injury which leads to chorioretinal rupture, vitreous hemorrhage, and fiberoglial proliferation.^1^ The findings of sclopetaria are bare sclera, vitreous hemorrhage, and intra‐retinal or subretinal hemorrhage. Despite severe retinal and choroidal injuries in chorioretinitis sclopetaria, retinal detachment does not usually occur due to spontaneous retinopexy and scar formation. Treatment for sclopetaria tends to follow two paths of management: surgery or observation. In cases with comorbidity, such as globe rupture or retinal detachment, surgery is indicated. Ludwig et al. showed that almost 40% of sclopetaria patients need immediate surgery.^1^ In our case, observation was the best choice regarding the intact globe, attached retina, and no visual impairment. Previous studies showed that almost 85% of patients with sclopetaria had a best‐corrected visual acuity of less than 20/20. This rate reaches 90% in patients who have concurrent IOFB. The primary location of the sclopetaria in our case was the inferonasal retina which is reported in nearly 18.6% of previous cases.^1^, ^2^

Intra‐orbital foreign body (IOFB) refers to the foreign body in the orbital cavity and outside the globe that can accompany open globe injury. Foreign bodies can be metallic (iron, aluminum, and lead), inorganic non‐metallic (plastic, glass, rock, and concrete), or organic (wood, thorns, and bones). The usual mechanism is high‐velocity projectile trauma such as gunshot injury, industrial injury, and hammer work. IOFB can be asymptomatic or lead to complications such as chorioretinal shock wave injury, decreased vision, double vision, optic neuropathy, cellulitis, and extraocular muscle damage.^3^, ^4^

**FIGURE 2:**
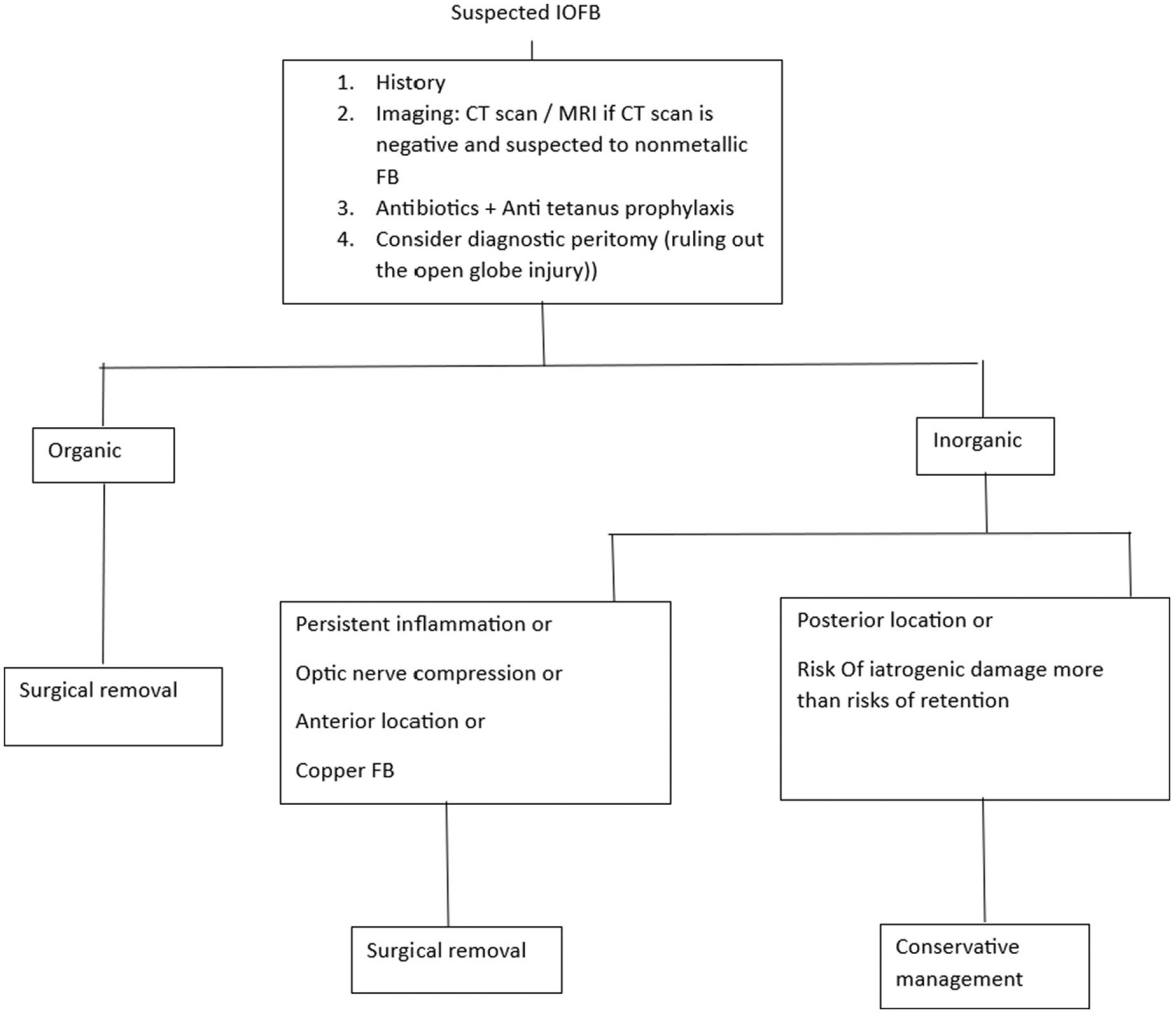
The flowchart diagram considering suspected IOFB cases.

Orbital CT scan is the gold standard imaging for detecting intra‐orbital foreign bodies in suspected patients with projectile trauma. Management depends on the types, size, and location of foreign bodies. Patients with Intra‐orbital foreign bodies should be given anti‐tetanus prophylaxis and broad‐spectrum antibiotics.^4^, ^5^ We summarized the management approach of the intra‐orbital foreign body in Figure 2.

## AUTHOR CONTRIBUTIONS

**Mehrdad Motamed Shariati:** Conceptualization; data curation; formal analysis; methodology; project administration; supervision; writing – review and editing. **Naser Sahraei:** Project administration. **Mahshid Sadeghi Kakhki:** Conceptualization; data curation; formal analysis; project administration; resources; writing – original draft.

## FUNDING INFORMATION

The authors received no funding.

## CONFLICT OF INTEREST STATEMENT

The authors declare that they have no competing interests.

## CONSENT

Written informed consent was obtained from the patient for the publication of this clinical image article.

## Data Availability

The data that support the findings of this study are available on request from the corresponding author. The data are not publicly available due to privacy or ethical restrictions.
